# Novel alendronate-CGS21680 conjugate reduces bone resorption and induces new bone formation in post-menopausal osteoporosis and inflammatory osteolysis mouse models

**DOI:** 10.1186/s13075-022-02961-0

**Published:** 2022-12-09

**Authors:** Ane Larrañaga-Vera, Kiran S. Toti, James S. Flatow, Alexandra J. Haraczy, Eugene Warnick, Harsha Rao, Zhan-Guo Gao, Sarah M. Sussman, Aranzazu Mediero, Philipp Leucht, Kenneth A. Jacobson, Bruce N. Cronstein

**Affiliations:** 1 Division of Translational Medicine, Department of Medicine, NYU Langone Health, 550 First Avenue, Medical Science Building, Room, New York, NY 251 USA; 2 Molecular Recognition Section, Laboratory of Bioorganic Chemistry, National Institute of Diabetes and Digestive and Kidney Diseases, National Institutes of Health, Bethesda, MD USA; 3 Medical College of Georgia at Augusta University, Augusta, GA USA; 4 Bone and Joint Research Unit, IIS-Fundación Jiménez Díaz UAM, Madrid, Spain; 5 Department of Orthopedic Surgery, New York University Langone Orthopedic Hospital, New York, USA; 6 Department of Cell Biology, New York University Grossman School of Medicine, New York, NY USA; 7 Division of Rheumatology, Department of Medicine, NYU Langone Health, New York, NY USA

**Keywords:** Osteoporosis, Inflammatory osteolysis, A_2a_ adenosine receptor, Bone formation, Bone resorption

## Abstract

Loss of bone is a common medical problem and, while it can be treated with available therapies, some of these therapies have critical side effects. We have previously demonstrated that CGS21680, a selective A_2A_ adenosine receptor agonist, prevents bone loss, but its on-target toxicities (hypotension, tachycardia) and frequent dosing requirements make it unusable in the clinic. We therefore generated a novel alendronate-CGS21680 conjugate (MRS7216), to target the agonist to bone where it remains for long periods thereby diminishing the frequency of administration and curtailing side effects. MRS7216 was synthesized from CGS21680 by sequential activation of the carboxylic acid moiety and reacting with an appropriate amino acid (PEG, alendronic acid) under basic conditions. MRS7216 was tested on C57BL/6J (WT) mice with established osteoporosis (OP) and WT or A2A KO mice with wear particle-induced inflammatory osteolysis (OL). Mice were treated weekly with MRS7216 (10mg/kg). Bone formation was studied after in vivo labeling with calcein/Alizarin Red, and μCT and histology analyses were performed. In addition, human primary osteoblasts and osteoclasts were cultured using bone marrow discarded after hip replacement. Receptor binding studies demonstrate that MRS7216 efficiently binds the A2A adenosine receptor. MRS7216-treated OP and OL mice had significant new bone formation and reduced bone loss compared to vehicle or alendronate-treated mice. Histological analysis showed that MRS7216 treatment significantly reduced osteoclast number and increased osteoblast number in murine models. Interestingly, cultured human osteoclast differentiation was inhibited, and osteoblast differentiation was stimulated by the compound indicating that MRS7216 conjugates represent a novel therapeutic approach to treat osteoporosis and osteolysis.

**Supplementary Information:**

The online version contains supplementary material available at 10.1186/s13075-022-02961-0.

## Introduction

Skeletal disorders are some of the most widespread health issues among the elderly population, impacting activities of daily life, greatly contributing to disability. According to the Bone and Joint Initiative, 19% of all health care visits are related to musculoskeletal disorders and constitute 5.8% Gross Domestic Product in the USA (https://www.usbji.org/programs/bmus).

Many skeletal conditions involve an imbalance between bone formation and destruction/remodeling, by osteoblasts and osteoclasts respectively that results in net bone loss [1, 2]. For example, osteoporosis, a condition in which bone formation slows over time, is characterized by low bone mass, increasing bone fragility and susceptibility to fracture (https://www.usbji.org/programs/bmus). Analysis of the NHANES database yields estimates that 7.8 million women and 2.3 million men in the USA are osteoporotic, and 10.5 million women and 3.3 million men in the USA could be affected by 2020 (https://www.usbji.org/programs/bmus).

Current treatments seek to diminish osteoclast activity, enhance osteoblast activity, or both. The most commonly used osteoporosis medications are bisphosphonates, which act as antiresorptives. Alendronate and zolendronate are currently the most cost-effective therapies [3]. Despite high success rates, these drugs are not ideal solutions. Long-term administration can cause side effects including femoral shaft or subtrochanteric fractures with atypical radiographic features, or osteonecrosis of the jaw [4–6]. Anabolic agents such as parathyroid hormone receptor agonists and antibodies directed against sclerostin have also been approved to treat osteoporosis. These drugs increase bone mineral density (BMD) by promoting new bone formation but, like bisphosphonates, have drawbacks. Parathyroid hormone receptor agonists can exacerbate bone resorption and they are not indicated for long-term use [7–9]. Anti-sclerostin antibodies are immunogenic in some patients and may increase risk of cardiovascular events [10–12].

Furthermore, none of the drugs discussed above has been proven effective against other bone pathologies involving inflammatory osteolysis, in which inflammation mediates osteoclast stimulation and inhibits osteoblast-mediated bone formation. Prosthetic wear particle-induced osteolysis can lead to loosening of prostheses, which causes 75% of arthroplasty revision surgeries, a problem projected to grow nearly 140% by 2030. Currently, there is no effective treatment, as bisphosphonates only partially prevent bone loss after joint-replacement surgery [12].

Mediero et al. showed that agonists of the A_2A_ adenosine receptor, a G-coupled protein receptor known for its anti-inflammatory proprieties [13], inhibit osteoclast differentiation and prevent bone osteolysis in wear particle-induced osteolysis models [14–16]. Additionally, stimulation of A_2A_ adenosine receptors induces bone formation through osteoblast activation enhancing healing in critical size fracture models [15, 17–25]. We therefore sought to design a small molecule inhibitor of bone resorption which also promotes new bone formation based on its activity at the adenosine A2A receptor. Because the A_2A_ adenosine receptor’s natural agonist, adenosine, has an extremely short half-life, various selective, specific agonists, such as CGS21680, have been designed. But despite promising results treating bone pathologies low solubility, side effects such as hypotension, and frequent dosing requirements preclude their clinical use to date [26, 27]. In this work, we have developed the novel compound, MRS7216, by linking alendronate to a CGS21680 molecule via a polyethylene glycol (PEG) linker which will localize to bone and retain its capacity to inhibit osteolysis and promote new bone formation. Since alendronate is well known to have a prolonged half-life in bone [28], we tested the hypothesis that MRS7216 could effectively treat bone pathologies such as osteoporosis and osteolysis due to its anabolic and anti-catabolic proprieties, while preventing the mechanism-related side effects of A_2A_ receptor agonists.

## Materials and methods

CGS21680.HCl was purchased from Tocris Bioscience (Minneapolis, MN, USA), and the PEG linkers were purchased from either Conju-Probe (San Diego, CA, USA) or BroadPharm (San Diego, CA, USA). All other chemicals and solvents (including anhydrous solvents) were from Sigma-Aldrich (St. Louis, MO, USA). Alendronate-CGS conjugate synthesis

### Synthesis of alendronate-CGS21680 conjugates

The synthesis began with the conversion of commercial A_2A_ receptor agonist CGS21680 1 to its active N-hydroxysuccinimide ester 2. This ester readily reacted with the primary amino group of alendronate to form conjugate 3 or with various functionalized linear PEG spacers to form terminal ester intermediate 6a or terminal carboxylate derivatives of varied chain length 7a–c, containing 6, 12, or 24 PEG units. Alternatively, compound 6a could be converted to compound 7a by ester hydrolysis. The final step leading to alendronate conjugates 8a–c with extended spacers consisted of condensation of the carboxyiate derivatives 7a–c with alendronate. The reference chain-extended alendronate derivatives 10a and 10b containing 6 or 12 PEG units were prepared by Boc protection of two terminal-amino PEG intermediates 5a and 5b, followed by condensation of the products 9a and 9b with alendronate and finally removal of the Boc group.

### Wear particle preparation

Ultra-high molecular weight polyethylene glycol particles (UHMWPE) (Sigma-Aldrich, St. Louis, MO, USA) were washed twice in 70% ethanol for 24 h at room temperature to prevent endotoxin contamination. The particles were then washed in phosphate-buffered saline (PBS; Sigma-Aldrich, St. Louis, MO, USA) and dried in a desiccator.

### Animal models

Mice were housed in a controlled environment, with a 12:12-h light-dark cycle, ad libitum access to water and food, and sacrificed in a CO_2_ chamber. A_2A_ adenosine receptor knockout (A2A KO) mice bred on a C57BL/6 background were kindly provided by Dr. Jiang Fan Chen. All protocols followed internationally recognized guidelines and were approved by the NYU SoM Institutional Animal Care and Use Committee.

Two animal models were used in this study, ovariectomy-induced osteoporotic mice (OP) [29], and UHMWPE-induced osteolytic mice (OL) [14]. Single animals were considered as experimental units, and sample size was calculated based on previously published data for each model. Multiple doses of AlenP and CGS21680 were tested in mice with UHMPWE-induced osteolysis for their effect on bone loss but none of the doses tested <10mg/kg/week had any effect on bone density in the affected areas (data not shown).

Fifteen ovariectomized mice and 5 age-matched WT mice were purchased from the Jackson Laboratory (Farmington, CT, USA). Surgery was performed on 6-week-old mice, as trabecular bone volume starts declining at this early age [30]. Beginning 6 weeks after surgery and continuing on a biweekly basis, animals were anesthetized and bone loss was evaluated by DXA scan using a PIXImus bone densitometer. Six weeks post-surgery, randomly allocated animals also began receiving weekly 10 mg/kg doses of MRS7216 (*n*=5), PEGylated alendronate (AlenP) (*n*=5), or saline (*n*=5) for 9 weeks. After IP injections, animals were monitored for 5 min for behavioral changes. Animals were sacrificed 9 weeks after surgery.

Osteolysis was induced by implantation of UHMWPE particles (Sigma-Aldrich, St. Louis, MO, USA) over the mouse calvaria, as previously described [14]. Briefly, 8-week-old male WT and A2A KO mice were anesthetized by intraperitoneal injection of 100 mg/kg ketamine and 10 mg/kg xylazine. All mice received a 0.5-cm incision over the calvaria. Ten mice received no particles, and the incision was closed without further intervention (healthy group). The remaining animals received 6mg of UHMWPE particles and were randomly distributed among groups. The 100 μL treatments were administered intraperitoneally (IP) as follows:

Beginning immediately before surgery and continuing on a weekly basis, 10 WT mice received 0.9% saline (Saline), 10 WT and 5 A2A KO mice received 10mg/kg of AlenP, and 10 WT and 5 A2A KO mice received 10 mg/kg MRS7216. Animals were sacrificed 14 days after surgery.

Bones of all animals were double labeled by IP injection of calcein (Sigma-Aldrich, St. Louis, MO, USA) 10 days before sacrifice and Alizarin Red Complexone (Sigma-Aldrich, St. Louis, MO, USA) 3 days before sacrifice.

### Micro-X-ray computed tomography (μCT) analysis

High-resolution μCT was used as previously described (18) to perform qualitative and quantitative analyses of resorbing areas in murine calvarial bone. Analyses were performed in the NYU College of Dentistry μCT core with a Skyscan 1172 μCT (Bruker, WI, USA) using 60kV, 167 μA, 9.7 micron pixel size, 2000×1332 matrix, 0.3 degree rotation steps, 6 averages, movement correction of 10, 0.5mm Al filter, 2 segments scanned per sample (56 min/segment). Images were reconstructed using the Skyscan NRECON software (histogram range 0–0.065, beam hardening correction of 35, Gaussian smoothing (factor 1, ring artifact correction of 7). For quantitative analysis of UHMWPE particle-induced osteolysis, a square-shaped region of interest across the parietal bone of approximately 4 mm right and left of the midline suture of the skull was placed in one of the 2D-reconstructed slices, as described previously (18), and ImageJ software was used to analyze calvarial bone resorption.

### Histomorphometry and histological studies

After sacrifice, bones were fixed in 4% PFA for 48 h. For double labeling, non-decalcified bones were submerged in 30% sucrose (Sigma-Aldrich, St. Louis, MO, USA), embedded in OCT medium (Fisher Scientific Hampton, NH, USA), and frozen. Sections of 10μm were cryosectioned and prepared using tape transfer method [31].

Other bones were fixed in 4% PFA for 48 h followed by 70% ethanol. They were then decalcified in 10% EDTA for 4 weeks. Paraffin-embedded histological sections (5 μm) were prepared and stained with TRAP staining or alkaline phosphatase immunohistochemistry.

After deparaffinization and acetate buffer incubation, samples were incubated in TRAP buffer for 30 min and counterstained with Fast Green.

For immunohistochemistry analysis, samples were deparaffinized and incubated in Proteinase K working solution (TE buffer (50mM Tris Base, 1mM EDTA, 0.5% Triton X-100), Proteinase K stock solution (Proteinase K, TE buffer, glycerol)) for 20 min at 37° C. Samples were then incubated in BLOXALL Blocking Solution (Vector Laboratories, Burlingame, CA, USA) for 30 min and overnight in primary antibody at 4° C. The next day, samples were incubated for 30 min in appropriate biotinylated secondary antibody and VECTASTAIN Elite ABC Reagent (Vector Laboratories, Burlingame, CA, USA). Finally, staining was performed with a DAB Substrate Kit (Abcam, Cambridge, UK) and counterstaining with hematoxylin.

Images were observed in a Leica (Morrisville, NC, USA) microscope equipped with SlidePath Digital Image Hub Version 3.0 software, or under a light microscope (Nikon, Melville, NY, USA) equipped with Nis Elements F3.0 SP7 software. Images were acquired with either a Nikon Eclipse fluorescence microscope or Leica SCN 4000 whole slide scanner. Analysis was performed using ImageJ software.

### Cell culture

Human bone marrow was obtained from 4 OA patients who underwent hip replacement surgery and all protocols were approved by the NYU School of Medicine’s IRB. Precursor cells were flushed out from femoral head with DMEM media with 10% fetal bovine serum (FBS), and 100 U/ml of penicillin and streptomycin (Lonza, Belgium) in sterile conditions and incubated for 48h non-adherent cells were collected and differentiated to osteoclasts and adherents cell were differentiated to osteoblasts as previously described [32].

Osteoclasts were differentiated for 7 days with 30 ng/mL RANKL in the presence of 0.001, 0.01, 0.1, 1, or 10μM alendronate (Sigma-Aldrich, St. Louis, MO, USA), AlenP, MRS7216 or MRS7216, and ZM241385 (Tocris, Bristol, UK) a selective A2A antagonist. Osteoclast differentiation was assessed by TRAP staining by incubating PFA fixed samples with TRAP buffer (0.1M acetate buffer, 0.3M sodium tartrate, 10mg/ml Naphthol AS-MX phosphate, 0.1% Triton X-100, 0.3mg/ml Fast Red Violet LB (Sigma-Aldrich, St. Louis, MO, USA)) for 10 min. Images were taken in a Leica (Morrisville, NC, USA) inverted microscope.

Osteoblasts were differentiated for 10 days with osteogenic medium (α-MEM (Lonza, Belgium) containing 1μM dexamethasone, 50 μg/mL ascorbic acid, 10mM β-glycerophosphate) in the presence of 1 μM AlenP, MRS7216 or MRS7216, and ZM241385. Consequently, cells were fixed in 4% paraformaldehyde and stained for 45 min with 2% Alizarin Red. The amount of mineral content was measured by eluting the Alizarin Red stain with 10% cetylpyridinium chloride and the optical density was measured at OD 570nm as previously described [32].

### Statistical analysis

Graphs were represented to reflect values for every single animal/patient and all the analysis were performed by blinded observers.

Statistical significance for differences between groups was determined with a one- or two-way ANOVA and Dunnett post hoc test. All statistics were calculated using GraphPad® software (GraphPad, San Diego, CA, USA).

## Results

### Synthesis of CGS21680-alendronate conjugates and their affinity for A2A adenosine receptor

**Fig. 1:**
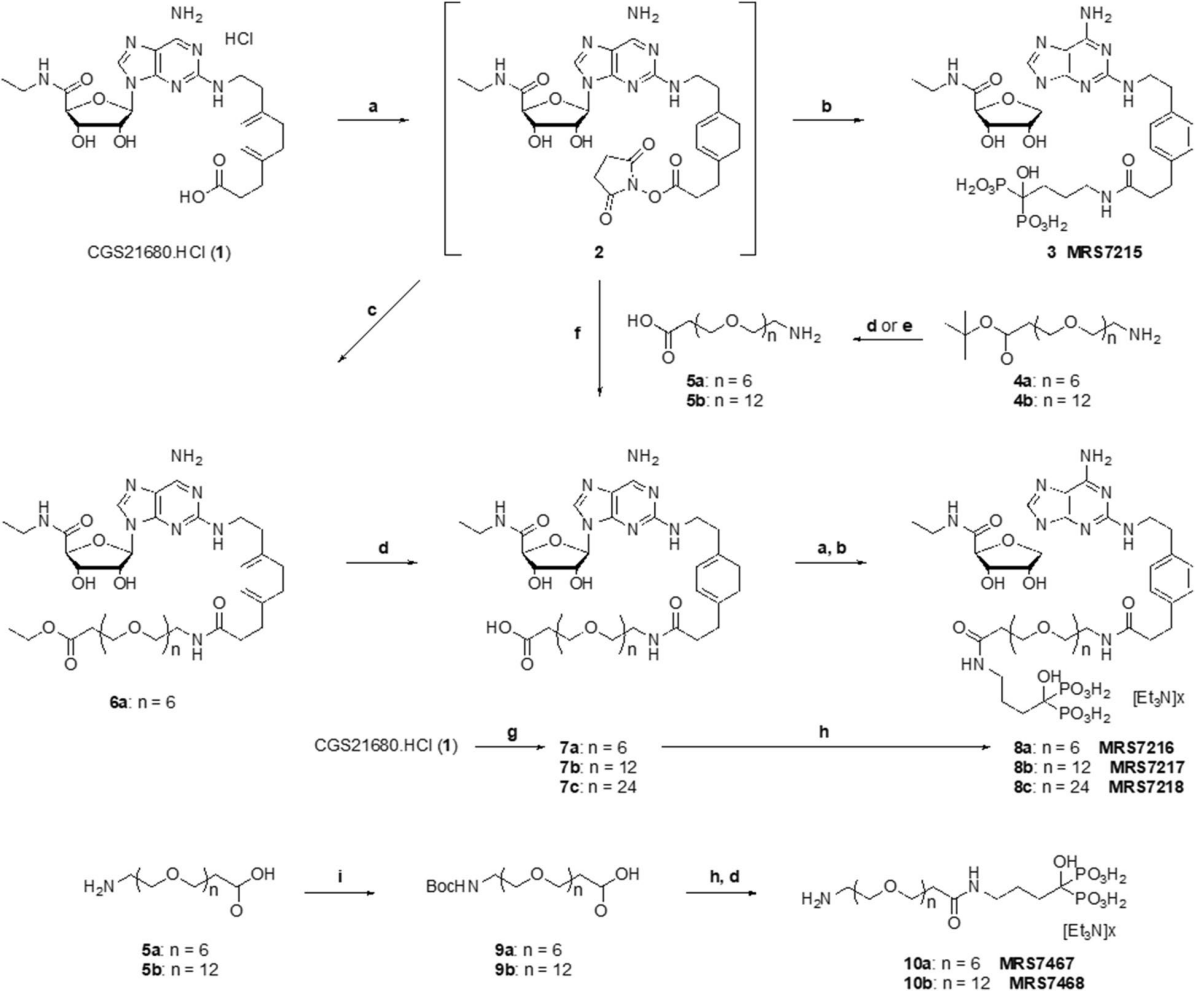
Chemical synthesis of alendronate conjugates of CGS21680 (3, 8a–8c) and the control alendronate functionalized congeners containing PEG (10a,b). Synthesis of PEGylated alendronic acid conjugates of A2A agonist CGS21680 (3, 8a,b,c) and its control compounds, PEGylated alendronic acids (10a,b). Reagents and conditions: (**a**) DMF, NHS, DIPEA, EDC.HCl, rt, 18h; (**b**) DIPEA, alendronic acid, H2O, rt, 18h, 6–22%; (**c**) DIPEA, NH2-PEG6-COOtBu, rt, 18h, 34%; (**d**) 1M HCl, 65 °C, 1h, 67%; (**e**) 30% TFA in DCM, 1h; quantitative (**f**) DIPEA, NH2-PEGn-COOH, H2O, rt, 18h, 50–62%; (**g**) (i) DMF, HATU, rt, 45min; (ii) DMF, DIPEA, NH2-PEG6-COOH.TFA, rt, 2h, 70–78%; (**h**) (i) DMF, HATU, rt, 45min; (ii) DMF, A [TBA]3, DIPEA, rt, 3h, 45–62%; (**i**) THF, DIPEA, (Boc)2O, rt, 18h, 73%

The target molecules (3, 8a–c; Fig. 1) were synthesized from CGS21680 (1), by sequential activation of the carboxylic acid moiety and reacting with the appropriate amino acid under basic conditions. The carboxylic acid group in 1 was activated by 1-ethyl-3-(3-dimethylaminopropyl) carbodiimide (EDC). HCl in the presence of N-hydroxysuccinimide to form the corresponding NHS-ester (2), which was reacted with alendronic acid to give MRS7215 (3) in a one-pot-two-step procedure. Incorporation of a PEG linker was explored following two different synthetic strategies: first, by reacting 2 with PEG6-t-butylcarboxylate 4a, followed by deprotection (34) using dilute hydrochloric acid to give 7a; and the second route by direct coupling of 2 with 5a. The later provided higher yields and was less laborious and was the method of choice to synthesize 7a–c. The carboxylic acid group in compounds having PEG6, 12, 24 was NHS-esterified using EDC.HCl and reacted with alendronic acid under basic conditions to afford the target molecule MRS7216 (8a), in moderate yields. Subsequently, different coupling conditions were compared for reaction scale-up (Table 1). Although COMU provided 7a in higher yield, this product failed to condense with alendronic acid. However, use of HATU as a coupling reagent proved comparatively fast, high yielding and economical to produce target molecule 8a. A direct synthesis of PEGylated alendronic acid 10a, by HATU activation of 5a as a hydrochloride salt and its coupling with TBA-alendronate, was unsuccessful. Hence, the amine group was converted to NH-Boc (9a,b) and the product condensed with alendronic acid, and subsequent deprotection of t-butyloxycarbonyl using 1N HCl at elevated temperature provided control alendronate derivatives 10a,b (AlenP).

**Table 1:** Comparison of the efficiency of three different coupling methods for the synthesis of MRS7216 8a.

Screening of coupling reagents and conditions			
Sr. No.	Solvents, Reagents, and conditions	1 → 7a	7a → 8a
**1.**	DMF, EDC.HCl, NHS, DIPEA, rt,18h, 5a/ alendronic acid (in H_2_O), rt, 18h	58%	6%
**2.**	DMF, HATU, DIPEA, rt, 45min, 5a/ A [TBA]_3_, rt, 3h	70%	45%
**3.**	DMF, COMU, DIPEA, rt, 45min, 5a/ A [TBA]_3_, rt, 3h	78%	-

A_2A_ agonist radioligand binding assay showed an affinity of MRS7216 K_i_=69.2 nM, which compared favorably with the affinity of parent agonist CGS21680, K_i_=21.5 nM. Both AlenP control molecules lacked evidence of A_2A_ adenosine receptor binding (Ki>10,000). In a cyclic AMP functional assay, we compared the activation by MRS7216 of the human A_2A_ and A_2B_ adenosine receptors expressed in HEK293 cells (Supl. Fig. 1). MRS7216 was a full agonist at the A_2A_ adenosine receptor with an EC_50_ of 9 nM. In the same assay, 1 μM CGS21680 was used as control. At the A_2B_ adenosine receptor, only 33% activation was produced by 10 μM MRS7216, compared to full agonist NECA (10 μM) as control.

### MRS7216 reverses osteoporotic bone loss in vivo

**Fig. 2:**
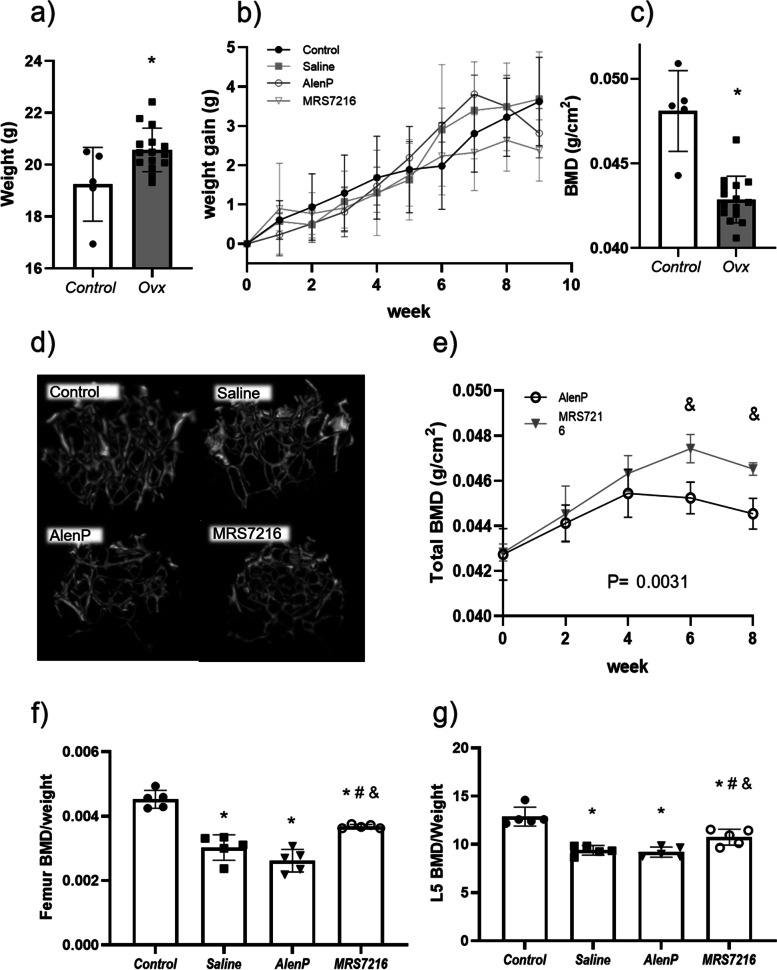
Characterization of osteoporotic mice show MRS7216 is effective at reversing osteoporosis. **a** Ovariectomy induced a significant weight increase in mice 6 weeks after the surgery. **b** After the treatments were administered all groups had a similar weight gain. **c** 6 weeks after the surgery Ovx mice had a 10% average decrease on BMD measured by DEXA scan. **d** Representative images of femur trabecular bone reconstruction by μCT. **e** Quantification of total body BMD on alendronate-PEG and MRS7216 treated mice measured by DEXA scan every 2 weeks. **f,g** Trabecular BMD representation of femur and L5 vertebra respectively. * *p*<0.05 vs Control, # *p*<0.05 vs Saline, & *p*<0.05 vs AlenP

We first tested the effect of MRS7216 in an established ovariectomy-induced OP mouse model. In this model, treatment with MRS7216, AlenP, or saline was administered weekly to mice starting 6 weeks after ovariectomy. MRS7216 or AlenP administration did not induce any obvious behavioral changes in mice compared to saline group. Ovariectomized mice (OVX) weighed significantly more than the control mice that did not have ovariectomy; however, there was no difference in the weight gain between groups (Fig. 2a,b). DXA scans of mice confirmed a significant reduction of bone mass in OVX mice 6 weeks after the surgery (*p* < 0.05). Subsequent biweekly scans indicated that weekly MRS7216 administration (Fig. 2e), but not saline or AlenP, significantly reversed OP bone loss after 6 weeks of treatment (Fig. 2d). At the study’s conclusion, following 9 weeks of treatment, mice were sacrificed and μCT scans showed significantly higher BMD in both femurs and L5 vertebrae for MRS7216-treated mice than mice that received saline or AlenP (*p* < 0.05) (Fig. 2d–g and Suppl. Tables 1, 2, 3). Because there was no enhancement of bone formation by the AlenP treatments, we concluded that enhanced bone formation from the A_2A_ adenosine receptor agonist likely caused the increase in BMD in this model. The disparity between the effects of AlenP on overall bone mineral density as opposed to its effects on femoral or lumbosacral bone mineral density is consistent with the disparate effects of alendronate on bone mineral density observed overall and in specific sites in patients (cf [33]).

### MRS7216 treatments reduce osteoclast number and increase osteoblast number in OP mice

**Fig. 3:**
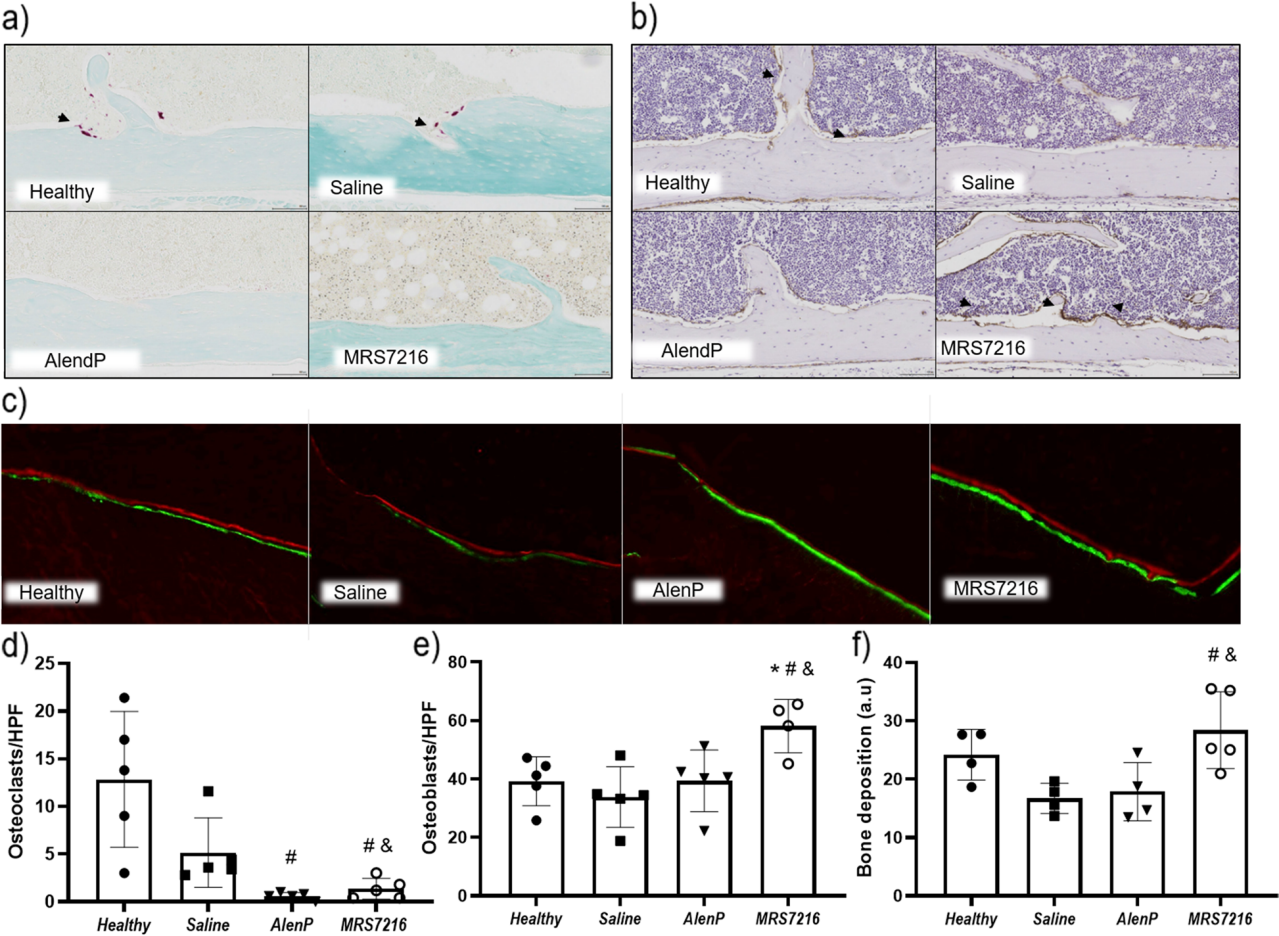
Histological studies of femurs from ovariectomized mice show a reduction in osteoclast number and an increase in osteoblast number and bone formation. **a–d** Representative images and quantification of TRAP staining of mice femur (in pink). **b–e** Representative images and quantification of ALP staining of mice femur (in brown). **c–f** Representative images and quantification of dynamic bone histomorphometry after double labeling with Alizarin Red and calcein. *p<0.05 vs Control, #*p*<0.05 vs Saline, & *p*<0.05 vs AlenP

To better understand how MRS7216 treatment enhances BMD, we first examined the effect of MRS7216, AlenP, and saline on bone formation in the femurs of these mice by use of fluorochrome labeling of new bone. Only MRS7216 stimulated an increase in new bone formation (Fig. 3c,f). This finding is consistent with the increased number of alkaline phosphatase positive osteoblasts in bone from mice treated with MRS7216 compared to either AlenP or saline (Fig. 3b,e). Both AlenP and MRS7216 treatment nearly completely eliminated TRAP+ osteoclasts in bone, as compared to mice treated with saline-treated mice (Fig. 3a,d). These results indicate that MRS7216, like AlenP, eliminates osteoclast formation and accumulation in bone but only MRS7216 is capable of stimulating an increase in osteoblasts in bone with a concomitant increase in new bone formation.

### MRS7216 prevents wear particle-induced bone osteolysis in vivo

**Fig. 4:**
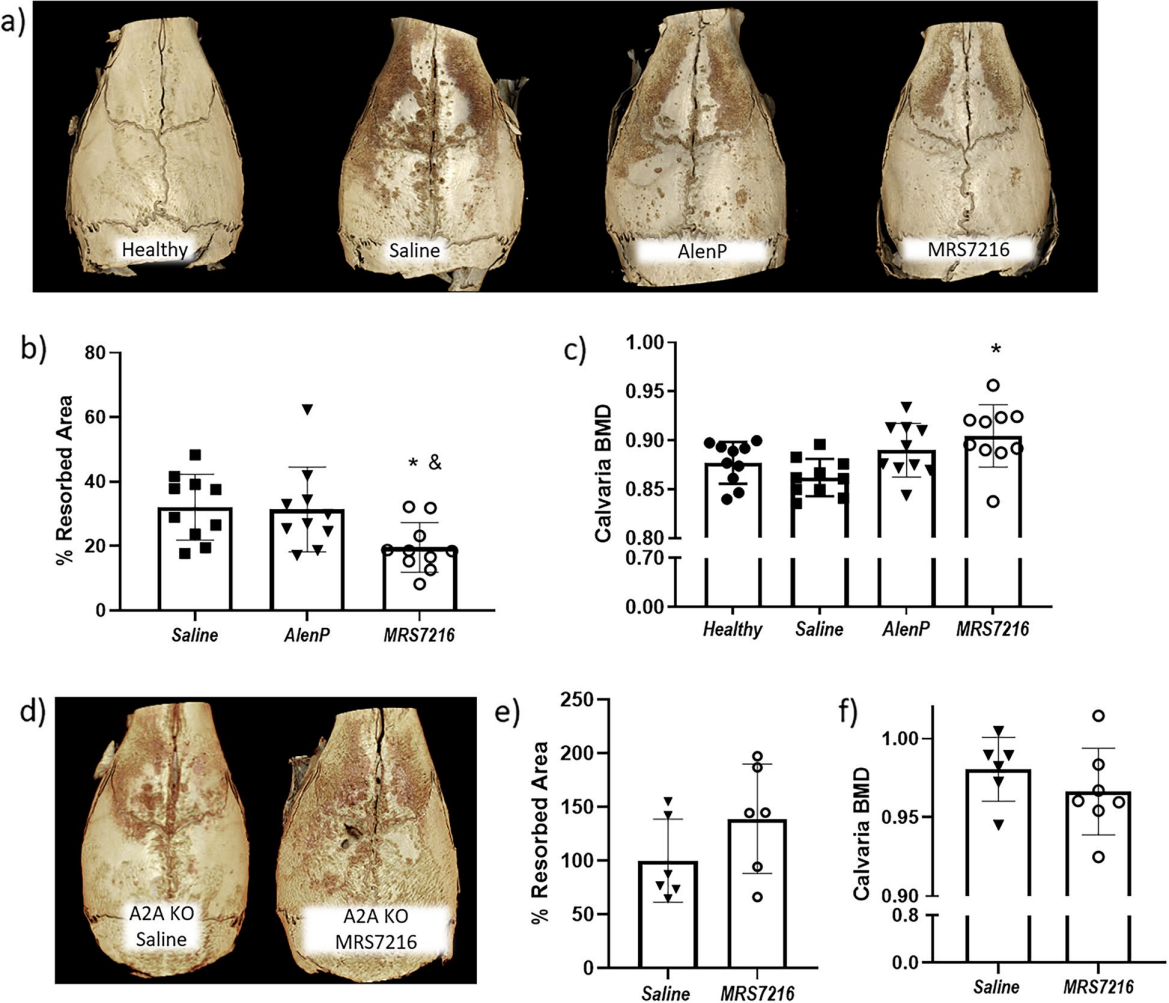
μCT studies of wear particle-induced osteolysis show how MRS7216 is effective at reducing bone pitting in WT mice but not in A2A KO mice. **a** Representative images of mice calvaria. **b** Quantification of the damaged bone area in WT mice calvaria. **c** BMD quantification in WT mice calvaria. **d** Quantification of the damaged bone area in A2A KO mice calvaria. **e** BMD quantification in A2A KO mice calvaria. * *p*<0.05 vs saline, & *p*<0.05 vs AlenP

A_2A_ adenosine receptor markedly diminishes inflammatory bone resorption due to wear particles (18), we therefore tested MRS7216 and AlenP on bone loss. Following implantation of UHMWPE, mice were treated with weekly doses of MRS7216, AlenP, or saline for 2 weeks an additional group did not receive particles was used as healthy control. MRS7216 or AlenP administration did not induce any behavioral changes in mice compared to saline group. After sacrifice, μCT analysis of mice calvariae showed a significant reduction in pitting and porosity after weekly MRS7216 treatment when compared with saline or AlenP mice. Inflammatory bone loss was similar in both AlenP- and saline-treated mice (Fig. 4b,c). To further demonstrate the specificity of MRS7216, for A_2A_ adenosine receptor, we tested the effect of MRS7216 in A_2A_ adenosine receptor KO mice and found that MRS7216 did not have a therapeutic effect (Fig. 4d,e).

### MRS7216 treatments reduce osteoclast and increase osteoblast number and bone formation in the inflammatory bone destruction model

**Fig. 5:**
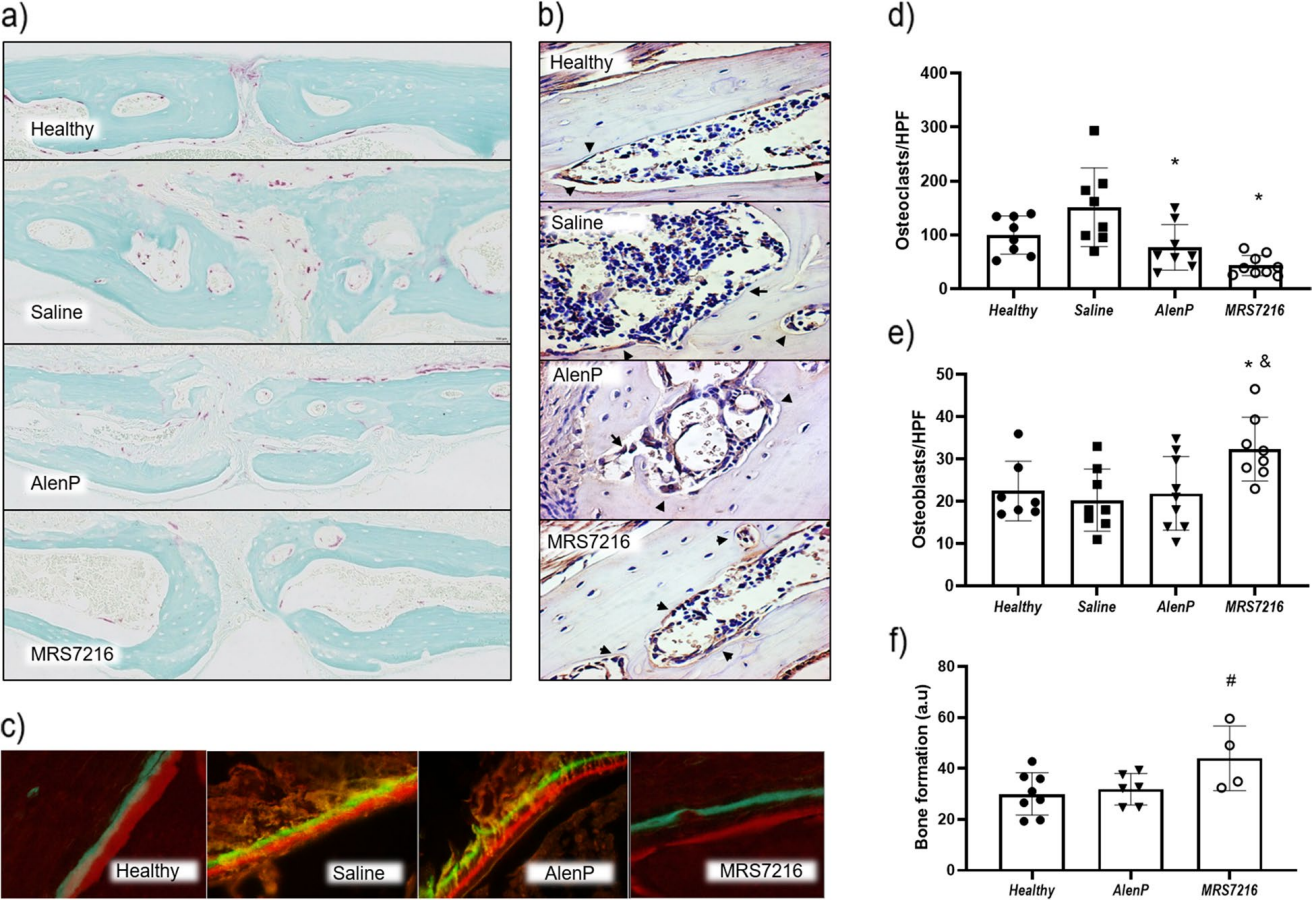
Histological studies of wear particle-induced osteolysis show a reduction in osteoclast number and an increase in osteoblast number and bone formation after MRS7216 treatment. **a–d** Representative images and quantification of TRAP staining of mice calvaria (in pink). **b–e** Representative images and quantification of ALP staining of mice calvaria (in brown). **c–f** Representative images and quantification of femur dynamic bone histomorphometry after double labeling with Alizarin Red and calcein. #*p*<0.05 vs healthy, *p<0.05 vs Saline, & *p*<0.05 vs AlenP

Histological studies of the OL mouse calvaria showed that AlenP- and MRS7216-reated mice had fewer TRAP+ osteoclasts in bone compared to those treated with saline alone (Fig. 5a,d). Similar to the findings in the osteoporosis model, immunohistochemistry of the calvaria showed more alkaline phosphatase positive osteoblasts in MRS7216-treated mice than healthy mice. In contrast, AlenP-treated mice did not have more osteoblasts than healthy mice (Fig. 5b,e). Consistent with the increase in osteoblasts, in vivo bone formation, measured following injection of the fluorochromes calcein and Alizarin, was enhanced in OL mice by MRS7216 (Fig. 5c,f).

### MRS7216 inhibits osteoclast formation and induces osteoblast mineralization in human primary cells

To determine whether the observed anti-osteoclastogenic and bone forming qualities could be translated to humans, we obtained primary human bone marrow cells and differentiated them towards osteoblast or osteoclasts following standard procedures.

**Fig. 6:**
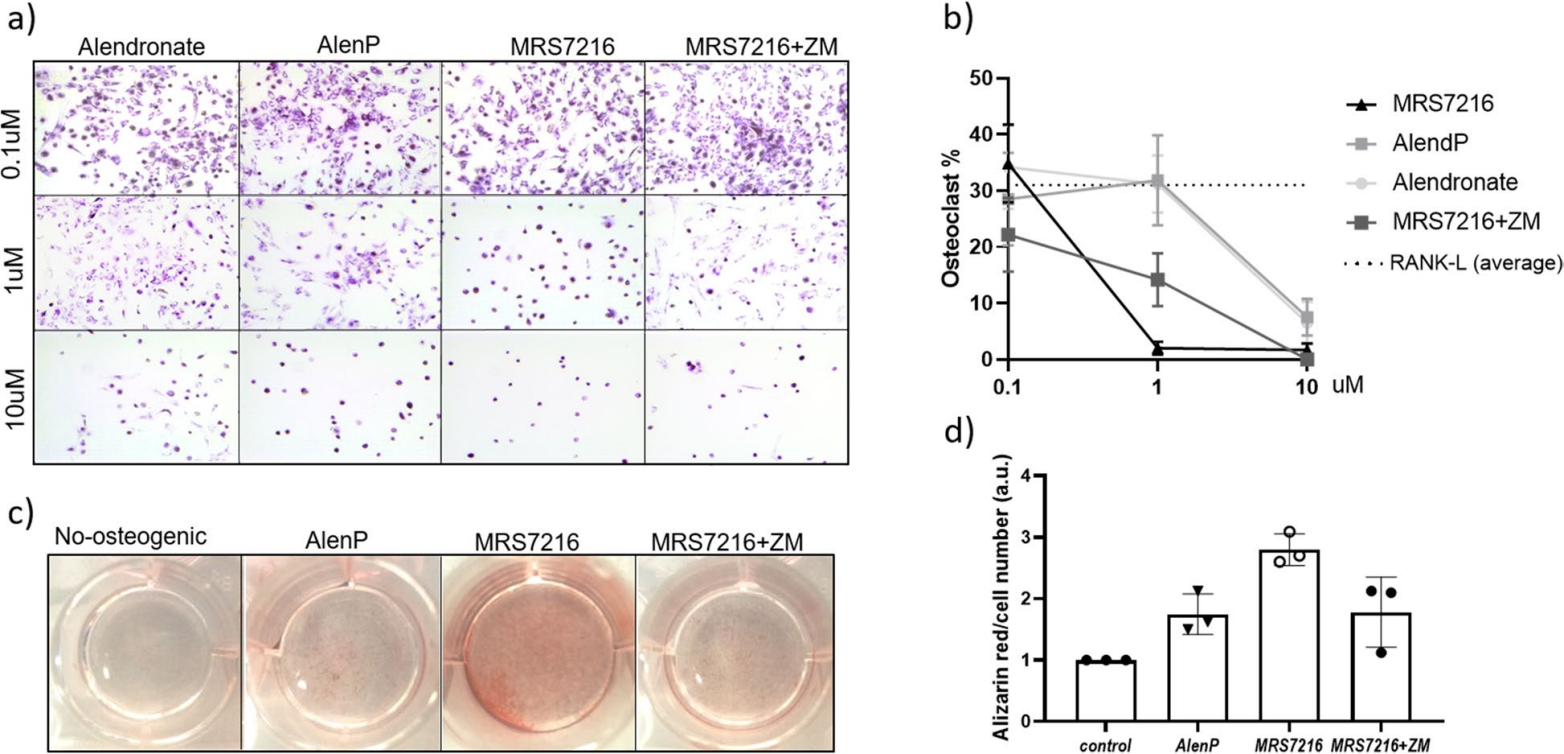
Cultured osteoclast and osteoblasts respond to MRS7216 treatment. **a,b** Representative images and quantification of differentiated osteoclast in response to different treatment doses. **p<0.05 vs AlenP and alendronate. **c,d** Representative images and quantification of the calcification on the osteoblast cultures in response to different treatments. * *p*<0.05 vs Control, # *p*<0.05 vs MRS7216+ZM, & *p*<0.05 vs AlenP

First, we tested if alendronate and AlenP had the same anti-osteoclastogenic proprieties and demonstrated that AlenP is as effective as alendronate (Fig. 6a,b). MRS7216 was significantly more effective at inhibiting osteoclast differentiation using 1μM dose, which has been described for its precursor CGS21680 (35), an effect that was partially reversed in the presence of an A_2A_ antagonist ZM21385, which, as we have previously demonstrated, increases osteoclast differentiation (Fig. 6a,b, [34, 35]), and is equally effective at higher doses.

Mineralizing capacity of MRS7216-treated osteoblasts was also enhanced in vitro as shown by the Alizarin Red staining (Fig. 6c,d). In contrast, AlenP-treated osteoblasts had the same mineralization rate as non-treated differentiated cells.

## Discussion

In this study, we developed a novel therapeutic candidate, MRS7216, a conjugate of alendronate, one the most commonly used anti-osteoporosis treatments [33], and CGS21680, a specific A_2A_ adenosine receptor agonist that has been previously described to have beneficial effect in bone [14, 34–37]. We report that the novel conjugate both halts bone resorption and stimulates new bone formation in ovariectomized mice, a model for post-menopausal osteoporosis. Surprisingly, treatment with AlenP increases overall bone density in the ovariectomy-induced osteoporosis model although AlenP did not increase bone density in either the femur or LS spine. Similar disparities in regional vs. total bone density changes have been found in patients treated with osteoporosis treated with alendronate [33]. Moreover, this same AlenP conjugate prevents inflammatory bone loss and promotes new bone formation in wear particle-induced bone destruction. Alendronate and AlenP showed the same capacity to inhibit osteoclastogenesis in both models of bone loss although alendronate appears to be more potent than AlenP. Based on the doses of alendronate required to inhibit osteoclastogenesis in vitro, it is likely that Pegylation of alendronate alters its pharmacokinetics. Nonetheless, only MRS7216 promotes new bone formation in the setting of both ovariectomy-induced and wear particle-induced bone loss. Furthermore, in vitro studies show that the conjugate can also affect human bone formation by increasing mineralization in vitro. Although we have not directly compared MRS7216 to other bone anabolic agents, the studies presented here suggest that MRS7216 is a potentially useful agent for the treatment of post-menopausal osteoporosis and wear particle-induced bone loss, the most common cause for loosening of orthopedic prostheses as weekly MRS7216 doses consistently improved UHMWP-induced bone lesions.

On a molar basis, there is nearly twice as much alendronate in AlenP as in MRS7261 (Molecular Weight 686 and 1268, respectively). Despite the increase in the molar quantity of alendronate present in AlenP as compared to MRS7261, there was no increase in the potency of the two compounds at doses less than 10mg/kg/week. At that dose level, both compounds reduced the number of osteoclasts in bone and increased overall bone mineral density. In contrast to MRS7216, AlenP did not affect either the number of osteoblasts in bone or bone deposition and there was no difference in osteoblast number or bone deposition in the AlenP- or saline-treated mice. Moreover, when studied in vitro, neither AlenP nor saline affected osteoblast differentiation or osteoid production. Thus, these findings are most consistent with the hypothesis that the difference in osteoblast number and bone deposition arises from the presence of the A2AR stimulation.

Ideally, a good therapeutic for osteoporosis and inflammatory bone disease should be easy to administer, low in dosing frequency, and free of side effects. In addition, there is no effective drug to prevent revision arthroplasties caused by inflammatory osteolysis. MRS7216 could be used to treat or prevent inflammatory osteolysis at the site of prosthesis placement and to treat osteoporosis effectively. Although we found no obvious toxicities of the AlenP treatment, more extensive studies should be carried out to better delineate the toxicity of this agent.

Because bisphosphonates are very frequently used to treat bone pathologies, several researchers have evaluated the use of alendronate or other bisphosphonates to treat wear particle-induced inflammatory osteolysis [12, 38–40], but despite common off-label use, their efficacy has not been proven. It has been hypothesized that the lack of efficacy of bisphosphonates in this setting might be due to the inflammatory mediators’ role in preventing osteoclast apoptosis [41, 42]. As one of the best described roles of the A_2A_ adenosine receptor is its function as an anti-inflammatory mediator [13], the use of MRS7216 conjugates that integrate antiresorptive and anti-inflammatory functions could overcome the limitations of bisphosphonates. This work has also shown that MRS7216 is more effective than PEGylated alendronate in reversing bone loss in an established murine osteoporosis model by preventing bone resorption and, more importantly, stimulating bone formation. Due to its capacity to stimulate bone formation MRS7216 could prevent some of the described side effects of alendronate, although that remains to be established.

In addition to alendronate, adenosine A_2A_ adenosine receptor agonists, such as CGS21680, have also shown promise as bone therapeutics on their own [43]. Recent studies indicate that estrogen regulates expression of CD73/CD39 in mice, ectoenzymes that hydrolyze ATP to adenosine in the extracellular space, and that following ovariectomy adenosine generation is diminished concomitantly with bone loss [44]. Moreover, the bone loss, in this model, was partially reversed by administration of an adenosine A_2B_ receptor agonist, the effect of A_2A_ adenosine receptor agonists was not tested in this work. Similarly, Takedachi and colleagues reported that overexpression of CD73 led to an increase in extracellular adenosine that promoted osteoblast differentiation [43]. Finally, by preventing formation of extracellular adenosine, tenofovir induces osteopenia in mice, which can be reversed by administration of dipyridamole, an agent that blocks purine uptake via ent1 and thereby increases extracellular adenosine [45]. This is clinically relevant since tenofovir induces osteopenia in patients as well.

A2A KO mice show a lower BMD than WT counterparts as well as an increased number of TRAP+ cells [16]. At the cellular level, Mediero et al. established that A_2A_ adenosine receptor agonism modulates osteoclast differentiation and prevents bone osteolysis in mice via PKA-dependent inhibition of NFκB nuclear translocation [34, 46, 47]. A_2A_ adenosine receptor agonists also have a role in bone formation. Both direct and indirect stimulation of A_2A_ adenosine receptors accelerates bone healing [15, 20, 35, 46, 48], an effect that is mediated by modulating osteoblast/osteoclast communication via regulation of Sema4D and Sema3A mediators to increase osteoblast activity and by the activation of canonical and non-canonical β-catenin pathways via PKA [34, 36, 37] in pre-osteoblasts, both well-established pathways promoting bone formation.

To date the major limitations for the use of A_2A_ adenosine receptor agonists as bone therapeutics are the side effects they cause. It has been reported that A_2A_ agonists cause vasodilation and trigger hypotension, intense locomotor depression, and core body temperature depression. We did not observe these toxicities upon acute administration of MRS7216. This might be related to the exclusion of MRS7216 to the periphery, as it is not expected to cross the blood brain barrier due to its molecular weight and polarity. Another source of this difference could be its biodistribution, which would be more restricted compared to small molecular weight A2A agonists. In addition, these side effects are not permanent and can last from several minutes to a few hours [49, 50]. So, the reduced dosing frequency of MRS7216 due to the extremely long half-life of alendronate in bone, the potent inhibition of inflammation and osteoclast formation, and stimulation of osteoblast-mediated bone formation make the bone-trophic A_2A_ adenosine receptor agonist MRS7216 conjugate an ideal candidate to use as a bone therapeutic. Once administered, alendronate disappears very rapidly from plasma, and the kidneys excrete the amount not taken up by bone tissues within 24 h. In contrast, the elimination of alendronate from bone tissue is slow, taking up to 12 years in humans [51]. The bone tropic nature of alendronate and, presumably, MRS7216 could help to diminish on target, A_2A_ adenosine receptor-mediated side effects. Moreover, MRS7216 could be administered with a lower frequency than other A_2A_ adenosine receptor agonists and unlike anabolic antibody treatments would not generate an immunogenic response.

The demonstration that an alendronate-A2AR agonist conjugate can both promote bone growth and inhibit inflammation in bone suggests potential uses for this type of agent that could be tested in future studies. Such an agent may be useful, for example, in the treatment of inflammatory arthritis-mediated bone damage. Prior studies have demonstrated that adenosine, acting at A2AR, promotes healing of critical fractures in bone [15, 19, 20, 25, 52] and administration of a similar bone-trophic A2AR agonist may increase the rate of fracture healing or stimulate bone healing in non-union of fractures. Future studies of these uses could lay the basis for these novel uses of alendronate-A2AR agonist conjugates.

## Conclusions

An adenosine A2A receptor agonist targeted to bone, MRS7216, reverses osteoporosis in a murine model of post-menopausal osteoporosis and prevents wear particle-induced osteolysis in mice. This agent may be a useful therapeutic that allows for the prolonged beneficial effects of A_2A_ adenosine receptor agonists and bisphosphonates in bone while preventing the side effects of the individual molecules. Moreover, because the A2A receptor agonist not only inhibits inflammation and bone resorption but stimulates new bone formation unlike other agents currently in use to treat osteoporosis and osteolysis.

## Supplementary Information

**Additional file 1. **Supplemental Data.

**Additional file 2. **Supplementary tables.

## Data Availability

All data and materials are available from the corresponding author.

## Abbreviations

A2AR: A_2A_ adenosine receptor
MRS7216: Alendronate-CGS21680 conjugate
BMD: Bone mineral density
WT: C57BL/6J
ESI: Electrospray ionization
FBS: Fetal bovine serum
OL: Inflammatory osteolysis
IP: Intraperitoneally
IE-LPLC: Ion-exchange chromatography
LPLC: Low-pressure liquid chromatography
μCT: Micro-X-ray computed tomography
OP: Osteoporosis
OVX: Ovariectomized mice
AlenP: PEGylated alendronate
PBS: Phosphate-buffered saline
PEG: Polyethylene glycol
TBAP: Tetrabutylammonium dihydrogen phosphate
UHMWPE: Ultra-high molecular weight polyethylene glycol particles
