# Supplementary material for: Novel alendronate-CGS21680 conjugate reduces bone resorption and induces new bone formation in post-menopausal osteoporosis and inflammatory osteolysis mouse models
Source: Arthritis Res Ther. 2022 Dec 9;24:265. doi: 10.1186/s13075-022-02961-0 (PMC9733060; doi:10.1186/s13075-022-02961-0)
Supplement: Supplementary file 1 — Additional file 1. Supplemental Data. [file 13075_2022_2961_MOESM1_ESM.docx]

Supplemental Data

CGS21680.HCl was purchased from Tocris Bioscience (Minneapolis, MN, USA) and the PEG linkers were purchased from either Conju-Probe (San Diego, CA, USA) or BroadPharm (San Diego, CA, USA). All other chemicals and solvents (including anhydrous solvents) were from Sigma-Aldrich (St. Louis, MO, USA). All reactions were carried out under nitrogen atmosphere using anhydrous solvents. Room temperature or rt refers to 25 ± 2 °C. NMR spectra were recorded on a Bruker 400 MHz spectrometer. Chemical shifts are given in ppm (δ), calibrated to the residual solvent signals or TMS and frequency calibrated by solvent for ^31^P NMR (D_2_O δ = 4.79; *X*i = 40.480742, H_3_PO_4_ external ; MestReNova 10.0.2). Exact mass measurements were performed on mass spectrometer equipped with a standard electrospray ionization (ESI) and modular LockSpray TM interface. The compounds were doubly purified, first by ion-exchange (low pressure liquid chromatography, LPLC) and then with C18-reverse phase-HPLC. The UV detection was set at 210, 254, 280 nm (fraction collection was triggered at 254 nm). C18-RP-HPLC did not show any peak that was detectable only at 210 nm, i.e. alendronic aicd or any salts/ non-UV active compounds. Ion-exchange chromatography (IE-LPLC) was performed using 10x100 mm Pharmacia column packed with GE Bioscience SOURCE 15Q resins, which was connected to Agilent 1100 HPLC system. The RP-HPLC was performed using Phenomenex Luna 5um C18(2)100A, AXIA, 21.2x250 mm column. Purity was determined using Agilent ZORBAX SB-Aq, 5um, 4.6x150 mm column, and a linear gradient of acetonitrile/ 5 mM tetrabutylammonium dihydrogen phosphate (TBAP) as mobile phase. There was no detectable presence of precursors in the doubly-purified product.

Abbreviations: A[TBA]_3_, tris(tetrabutylammonium)alendronate; COMU, (1-Cyano-2-ethoxy-2-oxoethylidenaminooxy)dimethylamino-morpholino-carbenium hexafluorophosphate; DCM, dichloromethane or methylenedichloride; DIPEA, *N*,*N*-diisopropylethylamine; DMF, Dimethyl formamide; EDC.HCl, 1-ethyl-3-(3-dimethylaminopropyl)carbodiimide hydrochloride; HATU, 1-[Bis(dimethylamino)methylene]-1H-1,2,3-triazolo[4,5-b]pyridinium 3-oxid hexafluorophosphate; HCl, hydrochloric acid; IE-LPLC, ion exchange low pressure liquid chromatography; NHS, *N*-hydroxy succinimide; RP-HPLC, reversed phase high pressure liquid chromatography; TEAA, triethylammonium aceate; TEAB, triethylammonium bicarbonate; TFA, trifluoroacetic acid.

Reagent Preparation: Tris(tetrabutylammonium)alendronate (**A[TBA]_3_**) was prepared by reacting aqueous alendronic acid (50 mg, 0.2 mmol in 4.0 mL water) and 1M aqueous tetrabutylammonium hydroxide (0.6 mL, 0.6 mmol) at 5°C. Water was removed by lyophilization and the residue used as a solution in anhydrous DMF.

CGS21680-NHS ESTER (**2**): Compound **1** (5.00 mg, 9.03 µmol) and N-hydroxysuccinimide (NHS, 2.15 mg, 18.66 µmol) were dissolved in anhydrous DMF (0.2 mL) at room temperature under inert atmosphere. Diisopropylethylamine (DIPEA, 3.3 µL, 18.66 µmol) and 1-ethyl-3-(3-dimethylaminopropyl)carbodiimide hydrochloride (EDC.HCl, 3.58 mg, 18.66 µmol) were added to the mixture as a solution in DMF (0.2 mL) at 0 °C. The reaction mixture was stirred overnight at room temperature by which time no starting material was present as indicated by high resolution mass analysis. This mixture was used as is for further reactions involving compound **2**. HRMS *m/z* [M + H]^+^ calculated for C_27_H_32_N_8_O_8_ 597.2421, found 597.2415.

CGS21680-Alendronic acid triethylammonium salt (**3, MRS7215**): To a flask containing the reaction mixture with compound **2** (9.03 µmol) at room temperature, DIPEA (16.0 µL, 93.30 µmol) and alendronic acid (4.65 mg, 18.66 µmol) were added; solid was dissolved with addition of H_2_O dropwise. Reaction mixture was stirred for 5h at room temperature. The solvents were evaporated, and the crude product was diluted in distilled water and purified using IE-LPLC (0 → 5 min, 100% H_2_O; 5 → 35 min, 100% H_2_O to 100% 1M triethylammonium bicarbonate (TEAB) buffer, liner gradient @flow rate of 3.0 mL/min). The compound eluting at 28 min was collected and lyophilized to give a white solid. The solid was dissolved in distilled water [**Note:** dilution by 0.1M TEAA may be needed to avoid peak splitting in HPLC due to different degree of salt formation] and purified further by RP-HPLC (10mM triethylammonium acetate (TEAA) buffer/acetonitrile 95/05 → 75/25 in 40 min, linear gradient @flow rate of 5.0 mL/min). The compound eluting at 38 minutes was collected and lyophilized to give a white solid. The solid was repeatedly dissolved in distilled water and lyophilized until the excess buffer was removed to afford 1.52 mg (22%) of final product **3** as a white solid. ^1^H NMR (400 MHz, D_2_O) δ 7.90 (s, 1H), 7.10 (d, *J* = 7.9 Hz, 2H), 7.03 (d, *J* = 7.9 Hz, 2H), 5.89 (d, *J* = 4.6 Hz, 1H), 4.89 (t, *J* = 5.0 Hz, 1H), 4.81 – 4.75 (m, 1H), 4.39 (d, *J* = 4.4 Hz, 1H), 3.56 (dt, *J* = 6.4, 13.3 Hz, 1H), 3.46 (dt, *J* = 6.0, 12.9 Hz, 1H), 3.26 (q, *J* = 7.3 Hz, 18H), 3.09 (q, *J* = 7.3 Hz, 2H), 3.03 (q, *J* = 7.3 Hz, 2H), 2.95 (t, *J* = 6.8 Hz, 2H), 2.78 (s, 3H), 2.73 (t, *J* = 7.1 Hz, 2H), 2.38 – 2.30 (m, 2H), 1.85 – 1.69 (m, 2H), 1.58 (d, *J* = 8.4 Hz, 3H), 1.17 (t, *J* = 7.2 Hz, 27H), 0.83 (t, *J* = 7.3 Hz, 3H). ^31^P NMR (162 MHz, D_2_O) δ 18.30. HRMS *m/z* [M - H]^-^ calculated for C_27_H_40_N_8_O_12_P_2_ 729.2163, found 729.2161. Purity – 96.66%

NH_2_-PEG_6_-COOH.TFA (**5a-TFA**): A sample of compound **4a** (30.72 mg, 75 µmol) in a flask was treated with a solution of Trifluoroacetic acid (TFA, 0.3 mL) in anhydrous CH_2_Cl_2_ (0.7 mL). The reaction was stirred at room temperature for 1h to give colorless liquid. Mass analysis indicated complete conversion of starting material to the desired product. Volatiles were evaporated and the excess TFA was chased by repeated evaporation with water. The residue was dissolved in water and lyophilized to afford **5a-TFA** as a colorless wax. The product was used as a solution in water or anhydrous DMF without further purification. HRMS *m/z* [H + M]^+^ calculated C_15_H_31_NO_8_ for 354.2128, found 354.2125.

NH_2_-PEG_6_-COOH.HCl (**5a-HCl**): A sample of compound **4a** (11.97 mg, 29 µmol) was treated with 1M HCl (0.2 mL) and stirred at 65°C for 1h. Mass analysis indicated complete conversion of starting material to the desired product. Volatiles were evaporated and the excess acid was chased by repeated evaporation with water. The residue was dissolved in water and lyophilized to afford **5a** as a colorless wax. The product was used as such without further purification and as a solution in water or anhydrous DMF. HRMS *m/z* [M + H]^+^ calculated for C_15_H_31_NO_8_ 354.2117, found 354.2122.

NH_2_-PEG_12_-COOH.HCl (**5b-HCl**): Following the procedure described for the synthesis of **5a-HCl**, a sample of compound **4b** (20 mg, 30 µmol) gave **5b-HCl** as a white solid. HRMS *m/z* [M + H]^+^ calculated for C_27_H_55_NO_14_ 618.3733, found 618.3730.

CGS21680-NH-PEG_6_-COOtBu (**6a**): To a flask containing the reaction mixture with compound **2** (9.03 µmol) at room temperature, compound **4a** (7 µL/ 7.35 mg, 18 µmol) was added and allowed to stir overnight. Solvent was evaporated under vacuum. The crude product was diluted by a solution of 25% distilled water in acetonitrile and purified using RP-HPLC (10mM TEAA/acetonitrile 80/20 → 40/60 in 40 min, linear gradient @flow rate of 5.0 mL/min**)**. The compound eluting at 36 min was collected and lyophilized to give compound **6a** (2.86 mg. 34%) as a white solid. ^1^H NMR (400 MHz, CD_3_OD) δ 7.98 (s, 1H), 7.14 (dd, *J* = 5.1, 7.5 Hz, 4H), 5.92 (d, *J* = 6.5 Hz, 1H), 4.97 (dd, *J* = 5.1, 6.4 Hz, 1H), 4.47 (dd, *J* = 2.8, 5.0 Hz, 1H), 4.39 (d, *J* = 2.8 Hz, 1H), 3.67 (t, *J* = 6.2 Hz, 3H), 3.64 – 3.52 (m, 28H), 3.45 (t, *J* = 5.5 Hz, 3H), 3.28 – 3.21 (m, 1H), 3.12 (dq, *J* = 7.2, 13.3 Hz, 1H), 2.91 – 2.80 (m, 6H), 2.50 – 2.41 (m, 5H), 1.43 (s, 11H), 1.03 (t, *J* = 7.3 Hz, 3H). HRMS *m/z* [M + H]^+^ calculated for C_42_H_66_N_8_O_13_ 891.4828, found 891.4832.

CGS21680-NH-PEG_6_-COOH (**7a**): Procedure – A: 1M HCl (0.2 mL) was added to compound **6a** (2.39 mg, 2.68 µmol) and stirred for 1h at 65 °C. The reaction mixture was subjected to rotary evaporation under vacuum and the crude product was diluted by 50% distilled water in acetonitrile and purified using RP-HPLC (10mM TEAA buffer/acetonitrile 95/05 → 65/35 in 40 min, linear gradient @flow rate of 5.0 mL/min). The compound eluting at 40 minutes was collected and lyophilized. The compound was repeatedly dissolved in water and lyophilized to remove residual buffer to give pure product as a white solid (1.50 mg, 67%). ^1^H NMR (400 MHz, D_2_O) δ 7.86 (s, 1H), 7.05 (d, *J* = 8.1 Hz, 2H), 7.00 (d, *J* = 8.2 Hz, 2H), 5.84 (d, *J* = 5.2 Hz, 1H), 4.82 (t, *J* = 5.2 Hz, 1H), 4.64 (dd, *J* = 4.0, 5.2 Hz, 2H), 4.37 (d, *J* = 4.0 Hz, 1H), 3.59 (t, *J* = 6.5 Hz, 2H), 3.49 (d, *J* = 2.7 Hz, 19H), 3.47 – 3.43 (m, 2H), 3.41 – 3.34 (m, 3H), 3.28 (t, *J* = 5.4 Hz, 2H), 3.12 (t, *J* = 5.2 Hz, 2H), 3.06 (t, *J* = 7.3 Hz, 1H), 2.99 (q, *J* = 7.2 Hz, 2H), 2.71 (t, *J* = 7.4 Hz, 5H), 2.36 (td, *J* = 5.4, 7.0 Hz, 5H), 1.15 (t, *J* = 7.3 Hz, 2H), 0.81 (t, *J* = 7.3 Hz, 3H). HRMS *m/z* [M + H]^+^ calculated for C_38_H_58_N_8_O_13_ 835.4202, found 835.4196.

Procedure – B: To a flask containing the reaction mixture with compound **2** (9.03 µmol) at room temperature, DIPEA (8.1 µL, 46.65 µmol) and compound **5a-TFA** (9.35 mg in 40 µL H_2_O, 20.00 µmol) were added and stirred at room temperature overnight. DMF was evaporated under reduced pressure and the crude product was diluted by a solution of 25% distilled water in acetonitrile and purified using the procedure described in Method-A to give **7a** (4.50 mg, 58%).

Procedure – C: CGS21680.HCl (5.00 mg, 9.03 µmol) was dissolved in anhydrous DMF (0.3 mL) under inert atmosphere, and to this was added DIPEA (3.2 µL, 18.06 µmol) followed by HATU (3.80 mg, 10.00 µmol). The reaction mixture was stirred at room temperature for 45 minutes. DIPEA (6.4 µL, 20.00 µmol) and **5a-TFA** (8.44 mg in 100 µL DMF, 18.06 µmol) were added sequentially and stirred at room temperature for 3h. DMF was evaporated under reduced pressure and the crude product was diluted by a solution of 25% distilled water in acetonitrile and purified using the procedure described in Method-A to give **7a** (5.29 mg, 70%).

Procedure – D: CGS21680.HCl (10.00 mg, 18 µmol) was dissolved in anhydrous DMF (0.5 mL) under inert atmosphere, and to this was added DIPEA (6.3 µL, 36 µmol) followed by COMU (10 mg, 21 µmol). The reaction mixture was stirred at room temperature for 45 minutes. DIPEA (12 µL, 40.00 µmol) and **5a-TFA** (13 mg in 300 µL DMF, 36 µmol) were added sequentially and stirred at room temperature for 3h. DMF was evaporated under reduced pressure and the crude product was diluted by a solution of 25% distilled water in acetonitrile and purified using the procedure described in Method-A to give **7a** (11.5 mg, 78%).

CGS21680-NH-PEG_12_-COOH (**7b**): To a flask containing the reaction mixture with Compound **2** (9.03 µmol) at room temperature, DIPEA (8.1 µL, 46.65 µmol) and Compound **5b-HCl** (13.13 mg in 80 µL H_2_O, 20.00 µmol) were added and stirred at room temperature overnight. DMF was rotary evaporated under vacuum and the crude product was diluted by a solution of 25% distilled water in acetonitrile and purified using RP-HPLC (10mM TEAA/acetonitrile 95/05 → 65/35 in 40 min, linear gradient @flow rate of 5.0 mL/min). The compound eluting at 43 min was collected and lyophilized. The compound was repeatedly dissolved in water and lyophilized to remove residual buffer to give pure product as a white solid (5.13 mg, 50%). ^1^H NMR (400 MHz, D_2_O) δ 7.84 (s, 1H), 7.06 (d, *J* = 8.1 Hz, 2H), 7.01 (d, *J* = 8.1 Hz, 2H), 5.84 (d, *J* = 5.2 Hz, 1H), 4.86 (t, *J* = 5.3 Hz, 1H), 4.65 – 4.62 (m, 1H), 4.36 (d, *J* = 3.8 Hz, 1H), 3.60 (t, *J* = 6.7 Hz, 2H), 3.58 – 3.46 (m, 47H), 3.45 (dd, *J* = 2.8, 5.8 Hz, 3H), 3.41 – 3.32 (m, 3H), 3.27 (t, *J* = 5.3 Hz, 2H), 3.12 (t, *J* = 5.3 Hz, 2H), 3.07 (q, *J* = 7.3 Hz, 3H), 2.99 (q, *J* = 7.2 Hz, 2H), 2.72 (t, *J* = 7.1 Hz, 4H), 2.35 (td, *J* = 2.4, 6.9 Hz, 5H), 1.15 (t, *J* = 7.3 Hz, 5H), 0.80 (t, *J* = 7.3 Hz, 3H). HRMS *m/z* [M + H]^+^ calculated for C_50_H_82_N_8_O_19_ 1099.5774, found 1099.5787.

Following Procedure – C described for the synthesis of **7a**, reacting CGS21680.HCl (20 mg, 0.036 mmol) with **5b**.TFA (53 mg, 0.072 mmol) gave **7b** (31 mg, 78%).

CGS21680-NH-PEG_24_-COOH (**7c**): To a flask containing the reaction mixture with compound **2** (18.06 µmol or 10 mg scale w.r.t. CGS21680.HCl) at room temperature, DIPEA (16 µL, 93.30 µmol) and NH_2_-PEG_24_-COOH (43.8 mg, 37.32 µmol) were added and stirred at room temperature overnight. DMF was evaporated under vacuum and the crude product was diluted by distilled water and purified using RP-HPLC (10mM TEAA/acetonitrile 80/20 → 60/40 in 40 min, linear gradient @flow rate of 5.0 mL/min). The compound eluting at 31 minutes was collected and lyophilized. The compound was repeatedly dissolved in water and lyophilized to remove residual buffer to give pure product as a white solid (18.71 mg, 62%). ^1^H NMR (400 MHz, D_2_O) δ 7.87 (s, 1H), 7.05 (q, *J* = 7.9 Hz, 4H), 5.86 (d, *J* = 5.4 Hz, 1H), 4.86 (t, *J* = 5.3 Hz, 1H), 4.63 (t, *J* = 4.6 Hz, 1H), 4.38 (d, *J* = 3.8 Hz, 1H), 3.79 – 3.43 (m, 113H), 3.43 – 3.34 (m, 3H), 3.31 (t, *J* = 5.3 Hz, 2H), 3.15 (t, *J* = 5.3 Hz, 2H), 3.09 (q, *J* = 7.3 Hz, 4H), 3.01 (qd, *J* = 2.6, 7.0 Hz, 2H), 2.73 (dd, *J* = 4.7, 9.7 Hz, 5H), 2.38 (td, *J* = 4.4, 7.0 Hz, 5H), 1.17 (t, *J* = 7.3 Hz, 6H), 0.82 (t, *J* = 7.3 Hz, 3H). HRMS *m/z* [M + H]^+^ calculated for C_74_H_130_N_8_O_31_ 1627.8920, found 1627.8903.

CGS21680-NH-PEG_6_-CO-alendronic acid triethylammonium salt (**8a, MRS7216**): Method – A: Compound **7a** (6.0 mg, 7.19 µmol) and NHS (1.66 mg, 14.4 µmol) were dissolved in DMF (0.2 mL) at room temperature. DIPEA (2.5 µL, 14.38 µmol) and EDC.HCl (2.76 mg, 14.38 µmol) were added as a solution in DMF (0.2 mL) at 0 °C. The reaction mixture was stirred overnight at room temperature. NHS ester formation was confirmed by HRMS *m/z* [M + H]^+^ calculated for C_42_H_61_N_9_O_15_ 932.4365, found 932.4365. DIPEA (12.51µL, 71.91 µmol) and alendronic acid (3.59 mg, 14.41 µmol) were added to the flask at room temperature; distilled water added dropwise to dissolve the solid. The reaction mixture was stirred at room temperature overnight. Solvents were evaporated and the crude product was diluted in distilled water and purified by IE-LPLC (0 → 5 min, 100% H_2_O; 5 → 35 min, 100% H_2_O to 100% 1M TEAB buffer, liner gradient @flow rate 3.0 mL/min). The compound eluting at 18 min was collected and lyophilized to give a white solid. The solid was dissolved in distilled water [**Note:** dilution by 0.1M TEAA may be needed to avoid peak splitting in HPLC due to different degree of salt formation] and placed for purification by RP-HPLC (10mM TEAA/acetonitrile 85/15 → 70/30 in 40 min, linear gradient @flow rate of 5.0 mL/min). The compound eluting at 30 minutes was collected and lyophilized to give final product **8a** as a white solid, which was repeatedly dissolved in water and lyophilized to remove excess TEAA buffer (0.62 mg, 6%). ^1^H NMR (400 MHz, D_2_O) δ 7.84 (s, 1H), 7.09 (d, *J* = 7.9 Hz, 2H), 7.03 (d, *J* = 7.9 Hz, 2H), 5.85 (d, *J* = 5.1 Hz, 1H), 4.90 (t, *J* = 5.2 Hz, 1H), 4.38 (d, *J* = 3.9 Hz, 1H), 3.60 (t, *J* = 6.3 Hz, 2H), 3.49 (d, *J* = 3.2 Hz, 17H), 3.45 (dd, *J* = 2.9, 5.8 Hz, 2H), 3.36 (dd, *J* = 3.1, 5.7 Hz, 2H), 3.26 (t, *J* = 5.2 Hz, 2H), 3.15 (q, *J* = 7.2 Hz, 12H), 3.07 (q, *J* = 7.3 Hz, 14H), 2.99 (q, *J* = 7.3 Hz, 2H), 2.73 (t, *J* = 6.9 Hz, 4H), 2.36 (t, *J* = 6.5 Hz, 4H), 1.80 (d, *J* = 13.3 Hz, 2H), 1.69 (s, 2H), 1.13 (dt, *J* = 7.3, 11.7 Hz, 38H), 0.79 (t, *J* = 7.3 Hz, 3H). ^31^P NMR (162 MHz, D_2_O) δ 18.25. HRMS *m/z* [M - H]^-^ calculated for C_42_H_69_N_9_O_19_P_2_1064.4107, found 1064.4089. Purity – 99.81%.

Method – B: To a mixture of compound **7a** (5.29 mg, 6.34 µmol) and DIPEA (2.2 µL, 12.64 µmol) in DMF (0.2 mL) was added HATU (2.90 mg, 7.61 µmol) at room temperature. After stirred for 45 minutes, tris(tetrabutylammonium)alendronate (15.40 mg, 12.67 µmol) and DIPEA (2.2 µL, 12.64 µmol) was added as a solution in DMF (67 µL) and stirred for 3h. Volatiles were evaporated under high vacuum and the crude product was dissolved in 0.1M TEAA buffer, centrifuged and the supernatant liquid purified following the procedure mentioned in method – A to get 3.64 mg (45%) of the desired product

CGS21680-NH-PEG_12_-CO-alendronic acid triethylammonium salt (**8b, MRS7217**): NHS ester formation of compound **7b** (5.13 mg, 4.67 µmol) and subsequent coupling with alendronic acid was achieved following a similar procedure described for the synthesis of compound **8a** (Method – A). The crude product **8b** was diluted in distilled water and IE-LPLC (0 → 5 min, 100% H_2_O; 5 → 35 min, 100% H_2_O to 100% 1M TEAB buffer, liner gradient @flow rate 3.0 mL/min). The compound eluting at 15 min was collected and lyophilized to give a white solid. The solid was dissolved in distilled water [**Note:** dilution by 0.1M TEAA may be needed to avoid peak splitting in HPLC due to different degree of salt formation] and placed for purification by RP-HPLC (10mM TEAA/acetonitrile 85/15 → 70/30 in 40 min, linear gradient @flow rate of 5.0 mL/min). The compound eluting at 38 minutes was collected and lyophilized to give a white solid which was repeatedly dissolved in water and lyophilized to remove excess TEAA buffer to afford 1.74 mg (26%) of final product **8b** as a white solid. ^1^H NMR (400 MHz, D_2_O) δ 7.85 (s, 1H), 7.11 (d, *J* = 8.1 Hz, 2H), 7.05 (d, *J* = 8.2 Hz, 2H), 5.87 (d, *J* = 5.2 Hz, 1H), 4.92 (t, *J* = 5.3 Hz, 1H), 4.39 (d, *J* = 3.9 Hz, 1H), 3.65 (t, *J* = 6.3 Hz, 2H), 3.61 – 3.49 (m, 48H), 3.49 – 3.45 (m, 2H), 3.42 – 3.35 (m, 3H), 3.29 (t, *J* = 5.3 Hz, 2H), 3.14 (d, *J* = 5.2 Hz, 2H), 3.09 (q, *J* = 7.3 Hz, 7H), 3.01 (q, *J* = 7.3 Hz, 2H), 2.75 (t, *J* = 7.3 Hz, 5H), 2.40 (dt, *J* = 6.7, 12.0 Hz, 5H), 1.92 – 1.76 (m, 3H), 1.71 (d, *J* = 7.0 Hz, 3H), 1.17 (t, *J* = 7.3 Hz, 8H), 0.81 (t, *J* = 7.3 Hz, 3H). ^31^P NMR (162 MHz, D_2_O) δ 18.17. HRMS *m/z* [M + H]^+^ calculated for C_54_H_93_N_9_O_25_P_2_ 1330.5836, found 1330.5853. Purity – 100%.

Following Procedure – B described for the synthesis of **8a**, reacting **7b** (31 mg, 0.028 mmol) with tris(tetrabutylammonium)alendronate (53 mg, 0.056 mmol) gave **8b** (28.17 mg, 65%).

CGS21680-NH-PEG_24_-CO-alendronic acid triethylammonium salt (**8c, MRS7218**): NHS ester formation of compound **7c** (18.71 mg, 11.50 µmol) and subsequent coupling with alendronic acid was achieved following a similar procedure described for the synthesis of compound **8a** (Method A). The crude product **8c** was diluted in distilled water and purified by IE-LPLC (0 → 5 min, 100% H_2_O; 5 → 35 min, 100% H_2_O to 100% 1M TEAB buffer, liner gradient @flow rate 3.0 mL/min). The compound eluting at 14 min was collected and lyophilized to give a white solid. The solid was dissolved in distilled water [**Note:** dilution by 0.1M TEAA may be needed to avoid peak splitting in HPLC due to different degree of salt formation] and placed for purification by RP-HPLC (10mM TEAA/acetonitrile 85/15 → 65/35 in 40 min, linear gradient @flow rate of 5.0 mL/min). The compound eluting at 37 minutes was collected and lyophilized to give a white solid which was repeatedly dissolved in water and lyophilized to remove excess TEAA buffer to afford 4.31 mg (20%) of final product **8c** as a white solid. ^1^H NMR (400 MHz, D_2_O) δ 7.87 (s, 1H), 7.19 – 6.98 (m, 4H), 5.87 (d, *J* = 5.2 Hz, 1H), 4.90 (t, *J* = 5.2 Hz, 1H), 4.39 (d, *J* = 3.8 Hz, 1H), 3.67 (t, *J* = 6.2 Hz, 2H), 3.64 – 3.49 (m, 91H), 3.47 (dd, *J* = 2.8, 6.0 Hz, 1H), 3.42 – 3.37 (m, 2H), 3.30 (t, *J* = 5.3 Hz, 2H), 3.17 – 3.05 (m, 10H), 3.01 (q, *J* = 7.3 Hz, 2H), 2.75 (t, *J* = 7.1 Hz, 4H), 2.40 (dt, *J* = 6.8, 14.5 Hz, 4H), 1.82 (dd, *J* = 9.8, 16.3 Hz, 2H), 1.77 – 1.64 (m, 2H), 1.17 (t, *J* = 7.2 Hz, 11H), 0.82 (t, *J* = 7.3 Hz, 3H). ^31^P NMR (162 MHz, D_2_O) δ 18.33. HRMS *m/z* [M - H]^-^ calculated for C_78_H_141_N_9_O_37_P_2_ 1856.8825, found 1856.8827. Purity – 99.89%.

BocNH-PEG_6_-COOH (**9a**): To a solution of compound **5a** (0.075 mmol) in THF (1 mL) was added DIPEA (40 µL, 0.225 mmol) and (Boc)_2_O (26 µL, 0.112 mmol). After stirring the reaction mixture for 18 h at room temperature, volatiles were evaporated and the residue purified by silica-gel column chromatography to afford **9a** as a colorless oil (25 mg, 73%, R_f_ = 0.45, TLC eluent 10% MeOH-CH_2_Cl_2_). ^1^H NMR (400 MHz, CDCl_3_) δ 5.15 (s, 1H), 3.78 (t, *J* = 6.2 Hz, 2H), 3.73 – 3.58 (m, 24H), 3.54 (td, *J* = 1.9, 5.2 Hz, 2H), 3.31 (q, *J* = 5.5 Hz, 2H), 2.75 (t, *J* = 6.3 Hz, 1H), 2.61 (t, *J* = 6.2 Hz, 1H), 1.45 (s, 9H). HRMS *m/z* [M + Na]^+^ calculated for C_20_H_39_N_1_O_10_ 476.2472, found 476.2467.

BocNH-PEG_12_-COOH (**9b**): Following the procedure described for the synthesis of **9a**, **5b** (0.062 mmol) gave **9b** as a colorless oil (30 mg, 68%, R_f_ = 0.35, TLC eluent 10% MeOH-CH_2_Cl_2_). ^1^H NMR (400 MHz, CDCl_3_) δ 5.16 (s, 1H), 3.78 (t, *J* = 6.1 Hz, 2H), 3.70 – 3.58 (m, 48H), 3.55 (t, *J* = 5.1 Hz, 2H), 3.32 (q, *J* = 5.4 Hz, 2H), 2.61 (t, *J* = 6.1 Hz, 2H), 1.45 (s, 9H). HRMS *m/z* [M + NH_4_]^+^ calculated for C_32_H_63_N_1_O_16_ 735.4491, found 735.4492.

NH_2_-PEG_6_-CO-alendronic acid triethylammonium salt (**10a, MRS7467**): Compound **9a** (25 mg, 0.055 mmol) was co-evaporated with anhydrous toluene (3 mL) and then dissolved in anhydrous DMF (0.5 mL). HATU (25 mg, 0,066 mmol) was added and stirred at room temperature for 45 min. A solution of [Bu_4_N^+^]_3_–alendronate in DMF (0.11 mmol) was added and stirred for 18h at room temperature. The formation of intermediate was confirmed by HRMS [M - H]^-^ calculated for C_24_H_50_N_2_O_16_P_2_ 683.2557, found 683.2567. The solvent was evaporated under vacuum and the residue was suspended in 1N HCl (3 mL) and stirred at 65 °C for 1 h. The volatile materials were evaporated under reduced pressure and the residue was neutralized with 1M triethylammonium bicarbonate buffer. The precipitates were filtered off and the filtrate lyophilized. The residue was dissolved in water and purified by RP-HPLC (10mM TEAA/acetonitrile 100/0 → 70/30 in 40 min, linear gradient @flow rate of 5.0 mL/min, collector UV = 210 nm). The compound eluting at 31 minutes was collected and lyophilized to give a white solid which was repeatedly dissolved in water and lyophilized to remove excess TEAA buffer to afford 23.5 mg (62%) of final product **10a** as a white solid. ^1^H NMR (400 MHz, D_2_O) δ 3.78 (q, *J* = 4.6, 5.4 Hz, 4H), 3.75 – 3.63 (m, 20H), 3.28 – 3.14 (m, 4H), 2.54 (t, *J* = 6.2 Hz, 2H), 2.01 – 1.86 (m, 2H), 1.82 (dt, *J* = 6.4, 9.5 Hz, 2H). ^31^P NMR (162 MHz, D_2_O) δ 18.38. HRMS *m/z* [M - H]^-^ calculated for C_19_H_42_N_2_O_14_P_2_ 583.2033, found 583.2035. Purity – 93.92% at 210nm.

NH_2_-PEG_12_-CO-alendronic acid triethylammonium salt (**10b, MRS7468**): Following the procedure described for the synthesis of **10a**, **9b** (30 mg, 0.042 mmol) gave **10b** as a colorless solid (21.4 mg, 54%; RP-HPLC, 10mM TEAA/acetonitrile 100/0 → 70/30 in 40 min, linear gradient @flow rate of 5.0 mL/min, collector UV = 210 nm, Rt = 37 min). ^1^H NMR (400 MHz, D_2_O) δ 3.89 (q, *J* = 4.7, 5.5 Hz, 4H), 3.86 – 3.72 (m, 40H), 3.40 – 3.22 (m, 4H), 2.64 (t, *J* = 6.2 Hz, 2H), 2.07 (dddd, *J* = 3.9, 7.7, 13.4, 17.0 Hz, 2H), 1.93 (dt, *J* = 6.5, 9.6 Hz, 2H). ^31^P NMR (162 MHz, D_2_O) δ 18.33. HRMS *m/z* [M - H]^-^ calculated for C_31_H_66_N_2_O_20_P_2_ 847.3606, found 847.3599. Purity - 90.56% at 210nm.

### Method for cyclic AMP assay

The alendronic acid conjugates were evaluated by a standard radioligand binding assay in membranes of HEK293 cells stably expressing the hA_2A_R subtype. The binding affinity was expressed as *K*_i_ value using the radioligand [^3^H]2-[*p*-(2-carboxyethyl)-phenethylamino]-5'-*N*-ethylcarboxamido adenosine ([^3^H]CGS21680, 10 nM) and calculated as previously reported (25). HEK293 cells expressing the human A_2A_ or A_2B_ receptors were seeded in 96-well plates and incubated in 100 µl medium at 37 °C overnight. The medium was removed the following day and cells were then treated with assay buffer containing rolipram (10 μM) and adenosine deaminase (3 units/ml) for 20 min followed by the addition of agonists, and then incubated for another 20 min. The reaction was terminated upon removal of the supernatant and addition of 100 µl Tween-20 (0.3%). Cyclic AMP content was measured using the ALPHA Screen cAMP assay kit as instructed by the manufacturer (PerkinElmer, Boston, MA).

### Table 1. Comparison of the efficiency of three different coupling methods for the synthesis of MRS7216 8a.

| **Screening of coupling reagents and conditions.** | | | |
| --- | --- | --- | --- |
| **Sr. No.** | Solvents, Reagents, and conditions | 1 → 7a | 7a → 8a |
| **1.** | DMF, EDC.HCl, NHS, DIPEA, rt,18h, 5a/ alendronic acid (in H_2_O), rt, 18h | 58% | 6% |
| **2.** | DMF, HATU, DIPEA, rt, 45min, 5a/ A[TBA]_3_, rt, 3h | 70% | 45% |
| **3.** | DMF, COMU, DIPEA, rt, 45min, 5a/ A[TBA]_3_, rt, 3h | 78% | - |
