# Supplementary material for: Novel alendronate-CGS21680 conjugate reduces bone resorption and induces new bone formation in post-menopausal osteoporosis and inflammatory osteolysis mouse models
Source: Arthritis Res Ther. 2022 Dec 9;24:265. doi: 10.1186/s13075-022-02961-0 (PMC9733060; doi:10.1186/s13075-022-02961-0)
Supplement: Supplementary file 2 — Additional file 2. Supplementary tables. [file 13075_2022_2961_MOESM2_ESM.docx]

## Supplementary tables

Femur Trabecular bone

|  | Control | Saline | Alendronate | MRS7216 |
| --- | --- | --- | --- | --- |
| BV/TV (%) | 3.113 ± 0.274 | 1.264 ± 0.209* | 0.771 ± 0.210* | 2.170 ± 0.347 * # & |
| BS/BV (1/mm) | 95.68 ± 2.76 | 98.33 ± 8.57 | 89.18 ± 4.61 | 100.03 ± 1.07 & |
| Tb.Th (μm) | 0.0444 ± 0.0015 | 0.0414 ± 0.0034 | 0.0468 ± 0.0014 # | 0.0401 ± 0.0014 * & |
| Tb.Sp (μm) | 0.321 ± 0.009 | 0.530 ± 0.061 * | 0.702 ± 0.100 * # | 0.443 ± 0.023 * & |
| Tb.N (1/mm) | 0.703 ± 0.071 | 0.307 ± 0.054 * | 0.164 ± 0.043 * # | 0.544 ± 0.099 * # & |
| Tb.Pf (1/mm) | 39.93 ± 1.59 | 39.03 ± 4.01 | 37.43 ± 2.48 | 36.37 ± 2.20 |

### Suppl. Table 1. µCT analysis of osteoporotic mice femoral, trabecular bones showing mean ± SEM. TV = total bone volume; BV = bone volume; BV/TV = bone volume/total volume; Tb. Th = trabecular thickness; Tb. Sp = trabecular space; Tb. N = trabecular number; TB.Pf trabecular bone pattern factor. *p  < 0.05. versus Control #p  < 0.05. versus Saline &p  < 0.05. versus Alendronate (ANOVA).

Femur Cortical bone

|  | Control | Saline | Alendronate | MRS7216 |
| --- | --- | --- | --- | --- |
| TV (mm^3) | 1.077 ± 0.075 | 1.192 ± 0.346 | 0.994 ± 0.042 | 1.017 ± 0.032 |
| BV (mm^3) | 0.523 ± 0.023 | 0.536 ± 0.160 * | 0.442 ± 0.014 * | 0.470 ± 0.010 * |
| BV/TV | 48.70 ± 2.03 | 44.92 ± 0.49 * | 44.50 ± 1.03 * | 46.26 ± 1.10 |

### Suppl. Table 2. µCT analysis of osteoporotic mice femoral, cortical bones showing mean ± SEM. TV = total bone volume; BV = bone volume; BV/TV = bone volume/total volume;

L5 vertebra Trabecular bone

|  | Control | Saline | Alendronate | MRS 7216 |
| --- | --- | --- | --- | --- |
| BV/TV (%) | 21.40 ± 2.45 | 14.95 ± 1.04 * | 12.34 ± 0.74 * | 16.11 ± 1.63 * & |
| BS/BV (1/mm) | 58.84 ± 0.75 | 67.54 ± 3.14* | 71.26 ± 2.80* | 68.78 ± 3.48* |
| Tb.Th (μm) | 0.056 ± 0.001 | 0.049 ± 0.002 * | 0.047 ± 0.002 * | 0.048 ± 0.002 * |
| Tb.Sp (μm) | 0.233 ± 0.018 | 0.279 ± 0.011 * | 0.298 ± 0.012 * | 0.254 ± 0.019 * # & |
| Tb.N (1/mm) | 3.84 ± 0.55 | 3.08 ± 0.18 * | 2.60 ± 0.21 * | 3.37 ± 0.32 & |
| Tb.Pf (1/mm) | 6.71 ± 2.51 | 10.46 ± 1.32 * | 13.73 ± 1.23 * # | 9.71 ± 1.44 & |

### Suppl. Table 3. µCT analysis of osteoporotic mice L5 vertebral bones showing mean ± SEM. TV = total bone volume; BV = bone volume; BV/TV = bone volume/total volume; Tb. Th = trabecular thickness; Tb. Sp = trabecular space; Tb. N = trabecular number; TB.Pf trabecular bone pattern factor. **p* < 0.05. versus Control ^#^*p* < 0.05. versus Saline ^&^*p* < 0.05. versus Alendronate (ANOVA).
